# Brain volumes and functional outcomes in children without cerebral palsy after therapeutic hypothermia for neonatal hypoxic‐ischaemic encephalopathy

**DOI:** 10.1111/dmcn.15369

**Published:** 2022-07-30

**Authors:** Arthur P. C. Spencer, Richard Lee‐Kelland, Jonathan C. W. Brooks, Sally Jary, James Tonks, Frances M. Cowan, Marianne Thoresen, Ela Chakkarapani

**Affiliations:** ^1^ Translational Health Sciences, Bristol Medical School University of Bristol Bristol UK; ^2^ Clinical Research and Imaging Centre University of Bristol Bristol UK; ^3^ School of Psychology University of East Anglia Norwich UK; ^4^ University of Exeter Medical School Exeter UK; ^5^ Department of Paediatrics Imperial College London London UK; ^6^ Faculty of Medicine, Institute of Basic Medical Sciences University of Oslo Oslo Norway; ^7^ Neonatal Intensive Care Unit, St Michael's Hospital University Hospitals Bristol and Weston NHS Foundation Trust Bristol UK

## Abstract

**Aim:**

To investigate whether brain volumes were reduced in children aged 6 to 8 years without cerebral palsy, who underwent therapeutic hypothermia for neonatal hypoxic‐ischaemic encephalopathy (patients), and matched controls, and to examine the relation between subcortical volumes and functional outcome.

**Method:**

We measured regional brain volumes in 31 patients and 32 controls (median age 7 years and 7 years 2 months respectively) from T1‐weighted magnetic resonance imaging (MRI). We assessed cognition using the Wechsler Intelligence Scales for Children, Fourth Edition and motor ability using the Movement Assessment Battery for Children, Second Edition (MABC‐2).

**Results:**

Patients had lower volume of whole‐brain grey matter, white matter, pallidi, hippocampi, and thalami than controls (false discovery rate‐corrected *p* < 0.05). Differences in subcortical grey‐matter volumes were not independent of total brain volume (TBV). In patients, hippocampal and thalamic volumes correlated with full‐scale IQ (hippocampi, *r* = 0.477, *p* = 0.010; thalami, *r* = 0.452, *p* = 0.016) and MABC‐2 total score (hippocampi, *r* = 0.526, *p* = 0.004; thalami, *r* = 0.505, *p* = 0.006) independent of age, sex, and TBV. No significant correlations were found in controls. In patients, cortical injury on neonatal MRI was associated with reduced volumes of hippocampi (*p* = 0.001), thalami (*p* = 0.002), grey matter (*p* = 0.015), and white matter (*p* = 0.013).

**Interpretation:**

Children who underwent therapeutic hypothermia have reduced whole‐brain grey and white‐matter volumes, with associations between hippocampal and thalamic volumes and functional outcomes.

## Graphical abstract

We investigated regional brain volumes in children aged 6–8 years without cerebral palsy, cooled for neonatal hypoxic‐ischaemic encephalopathy (patients) and matched controls. Patients had reduced volumes of whole‐brain grey matter and white matter, and reduced volumes of pallidi, hippocampi, and thalami not independent of total brain volume. In patients, cognitive and motor scores correlated with the volumes of the hippocampi and thalami, independent of age, sex and total brain volume.

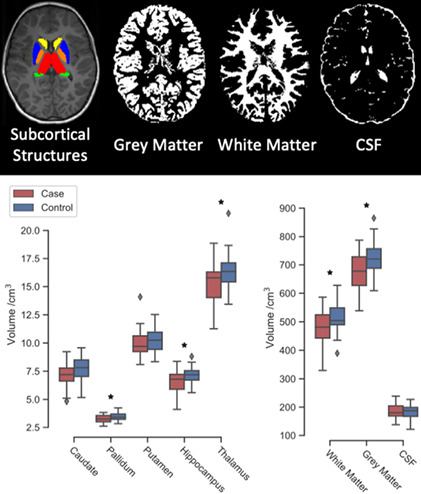

**Abbreviations:**
BGT: basal ganglia and thalami
HIE: hypoxic‐ischaemic encephalopathy
MABC‐2: Movement Assessment Battery for Children, Second Edition
PLIC: posterior limb of internal capsule
TBV: total brain volume

What this paper adds

- Patients who underwent therapeutic hypothermia for neonatal hypoxic‐ischaemic encephalopathy had lower whole‐brain white‐ and grey‐matter volumes than controls.
- These patients had smaller pallidi, hippocampi, and thalami.
- Subcortical volume differences were not independent of total brain volume.
- Cognitive and motor scores correlated with hippocampal and thalamic volumes in the patients.

Therapeutic hypothermia is now standard care for neonatal hypoxic‐ischaemic encephalopathy (HIE) secondary to perinatal asphyxia in high‐income countries and has been shown to reduce the risk of death and severe disability compared with normothermia management.^1^ Studies have reported that 74% to 90% of those who undergo therapeutic hypothermia survive past 18 months, and 73% to 85% of these do not have cerebral palsy (CP).^2^, ^3^, ^4^ Follow‐up assessment confirmed that 79% of survivors do not have CP at 6 to 7 years.^5^, ^6^ In children without CP, nearly 21% had cognitive impairments and 24% had high risk of motor impairment at 6 to 8 years.^7^

Despite the benefits of therapeutic hypothermia, we have previously demonstrated that early school‐age children who underwent therapeutic hypothermia for HIE and did not develop CP have altered brain structural connectivity^8^, ^9^ and reduced cognitive, motor, and behavioural scores^7^, ^10^, ^11^ compared with typically developing controls matched for age, sex, and socioeconomic status. In addition, motor difficulties were not predicted from developmental scores assessed at 18 months of age using the Bayley Scales of Infant and Toddler assessment.^11^

It is well established that the acute hypoxic‐ischaemic insult preceding HIE leads to characteristic patterns of brain injury on neonatal magnetic resonance imaging (MRI) scans, particularly to the deep grey‐matter structures including the hippocampus, thalamus, and basal ganglia.^12^ In children and adults who had mild to moderate HIE, before widespread use of therapeutic hypothermia, hippocampal^13^ and total brain and cortical volumes^14^ were found to be reduced when compared with controls. Injury to subcortical structures in neonates with HIE was shown to be associated with adverse neurodevelopmental outcomes.^15^, ^16^

It is not known whether children who have been treated with therapeutic hypothermia for HIE and who do not develop CP have lower volumes of subcortical structures and whether these subcortical volumes are associated with motor and cognitive outcomes. Therefore, we compared the volumes of brain tissues and subcortical structures between early school‐age children cooled for HIE, without CP, and control children matched for age, sex, and socioeconomic status. Second, we examined the relation between subcortical volumes and motor and cognitive outcomes. Finally, we investigated the association between qualitative scores of regional brain injury on neonatal MRI and regional brain volumes at early school‐age.

## METHOD

### Participants

This study was conducted at the Clinical Research and Imaging Centre (CRiCBristol), University of Bristol, UK, with approval from the North Bristol Research Ethics Committee (REC ID 15/SW/0148). Participants' assent was ensured at all times and informed and written consent was obtained from the parents of participants. Patients were sequentially selected from the cohort of children who received therapeutic hypothermia between October 2007 and November 2012 under a standard protocol for perinatal asphyxia‐induced moderate to severe encephalopathy confirmed by amplitude‐integrated electroencephalogram (EEG) assessment.^17^ These data are maintained by the Bristol Neonatal Neurosciences group at St Michael's Hospital, Bristol, UK, under previous ethics approval (REC ID 09/H0106/3).

#### Patients

Patients were aged 6 to 8 years and did not have a diagnosis of CP at 2 years based on assessment of motor function and neurological examination (all assessed by the same experienced clinician). Absence of CP at 6 to 8 years was confirmed using a standard clinical neurological examination including assessment of tone, motor function, and deep tendon reflexes. We excluded children who (1) were cooled outside the standard criteria; (2) were born before 35 weeks' gestation; (3) had an additional diagnosis, for example a metabolic disorder; or (4) did not have English as their primary spoken language.

Patients underwent neonatal MRI, which was qualitatively assessed, by an experienced perinatal neurologist (FC) for the presence and extent of brain injury. This was quantified, in the basal ganglia and thalami (BGT), white matter, and cortex (each on a score of 0–3), and the posterior limb of internal capsule (PLIC) (score 0–2), where a higher number indicates more severe injury.^17^, ^18^ Criteria for these scores are described in Table S1.

#### Controls

We recruited controls from schools around Bristol. Schools were excluded where a patient was currently attending, to protect participants' confidentiality. We included children who were born after 35 weeks' gestation, had not had perinatal asphyxia with HIE, and spoke English as their primary spoken language. To minimize the chance of recruiting children whose parents had concerns (e.g. about their development), parents were made aware that results of children's individual assessments would not be made available to them.

Socioeconomic status was assessed using the index of multiple deprivation as defined for England by the UK Government (www.gov.uk/government/statistics/english‐indices‐of‐deprivation‐2019). The 1 to 10 scale (where 10% of neighbourhoods are assigned to each number) is determined for a given neighbourhood from seven domains of deprivation including income, employment, education, health, crime, barriers to housing and services, and living environment, indicating the decile within which the local area is ranked in the country, from most deprived (1) to least deprived (10). We assessed the index of multiple deprivation for each participant on the basis of postcode at birth.

### Cognitive and motor assessments

Psychologists (led by JT), blinded to patient–control status, assessed the cognitive abilities of the children using the Wechsler Intelligence Scale for Children, Fourth Edition^19^ including subscales of working memory, processing speed, verbal comprehension, and perceptual reasoning from which full‐scale IQ is derived, which has a normative mean (SD) of 100 (15). Two researchers (RL‐K and SJ) assessed motor ability using the Movement Assessment Battery for Children, Second Edition (MABC‐2).^20^ Each assessment was videotaped and reviewed, and scoring agreed by consensus. The MABC‐2 has three subscale (manual dexterity, aiming and catching, and balance) standard scores which are combined to give an MABC‐2 total score, which has a normative mean (SD) of 10 (3). This score indicates high‐risk for, or at risk of, motor impairment at the 5th or 15th centiles respectively.

### MRI acquisition

All children were scanned using a 3 tesla Siemens Magnetom Skyra (Munich, Germany) and a receive‐only 32‐channel head coil. Following acquisition of localizer images, a T1‐weighted volumetric scan was obtained with the magnetization‐prepared rapid acquisition gradient echo sequence using the following parameters: echo time 2.19 ms; inversion time 800 ms; repetition time 1500 ms; flip angle 9°; field of view 234 mm × 250 mm; 176 slices; 1.0 mm isotropic voxels; generalized autocalibrating partially parallel acquisitions acceleration factor 4.^21^ Volumetric data were quality controlled by assessors blinded to patient–control status (JCWB and APCS) and scans with excessive movement artefact excluded.

A brain tissue mask was created for each participant's T1‐weighted data using either SPM8‐VBM (http://fil.ion.ucl.ac.uk/spm)^22^ or the CAT12 module in SPM12 (http://www.neuro.uni‐jena.de/cat)^23^ depending on which gave better delineation of the brain surface. Subsequently, the brain was segmented into grey matter, white matter, and cerebrospinal fluid using the automated tissue type segmentation tool, FAST,^24^ from the FMRIB software library (FSL, https://fsl.fmrib.ox.ac.uk/fsl), to obtain the whole‐brain volume of each tissue type. Whole‐brain grey‐matter volume included both cortical and subcortical grey matter. Total brain volume (TBV) was then calculated as the sum of grey‐ and white‐matter volumes.

**FIGURE 1:**
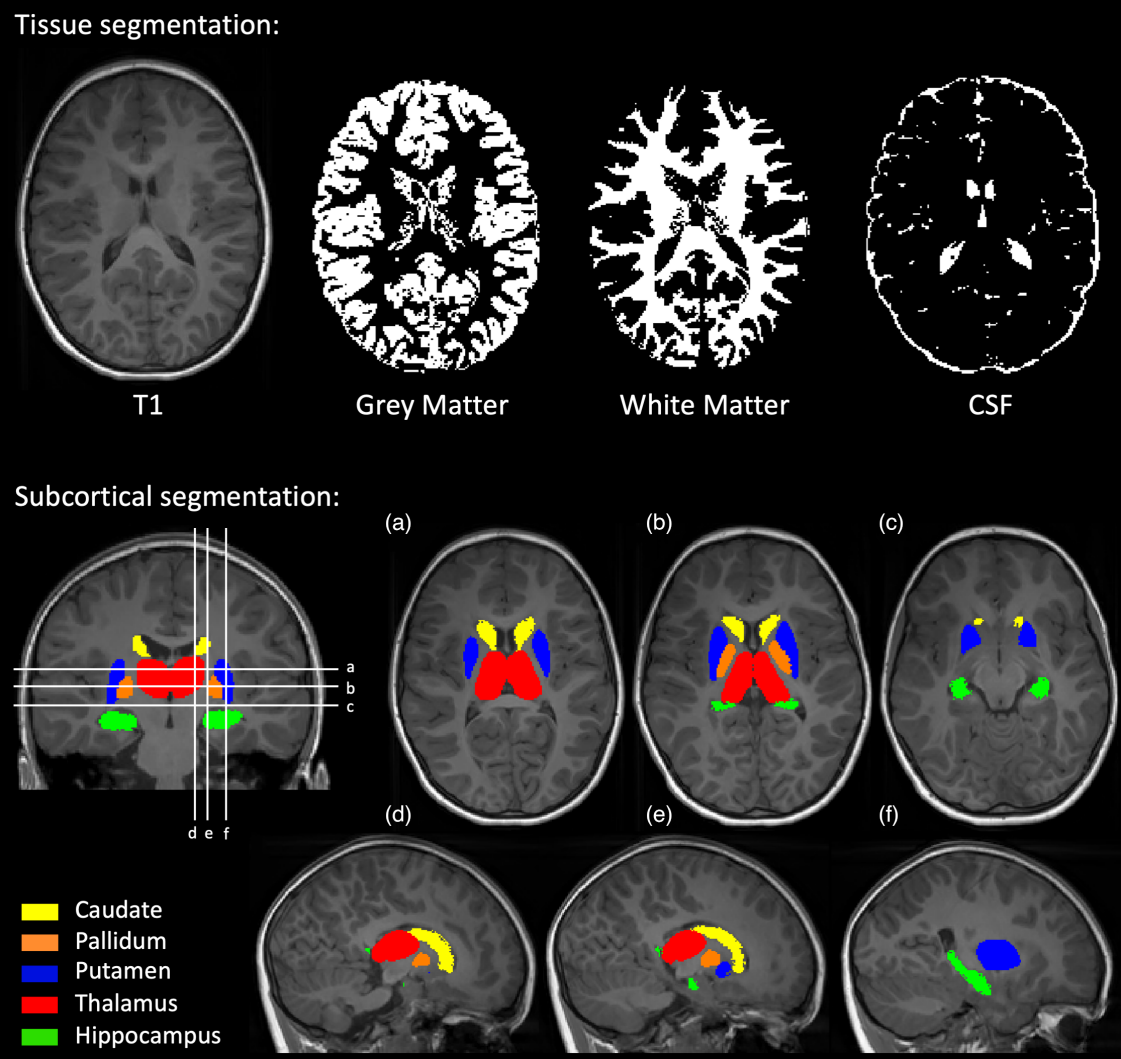
Segmentation of T1‐weighted images. Volumes of grey matter, white matter, and cerebrospinal fluid (CSF) were measured using the FAST automated tissue type segmentation tool. Volumes of subcortical structures were measured using the FIRST automated subcortical segmentation tool. Abbreviation: CSF, cerebrospinal fluid.

Subcortical grey‐matter structures, including the basal ganglia (caudate, pallidum, and putamen), hippocampus, and thalamus, were segmented from each individual's T1‐weighted scan using FSL's automated subcortical segmentation tool, FIRST,^25^ to obtain their volumes. Upon visual inspection of the masks of subcortical structures produced by FIRST, and masks of tissue types produced by FAST, no manual correction for delineation of structures was found necessary. As hypoxic brain injury is a brain‐wide insult, we expected to see bilateral changes. Therefore, to reduce the effect of multiple comparisons, for each subcortical structure we summed the left and right volumes to give the total bilateral volume. Figure 1 gives a demonstration of segmentation of tissue types and subcortical structures.

### Statistical analysis

Normality was tested with a Q–Q plot and Shapiro–Wilk test. Grey matter, white matter, cerebrospinal fluid, and subcortical grey‐matter volumes were compared between the patient and control groups using a two‐tailed *t*‐test as these variables were normally distributed. One‐way analysis of covariance (ANCOVA) was then used to compare subcortical volumes between groups while controlling for TBV. To examine the association between regional brain volumes and functional outcome, we then assessed the partial Pearson correlation between subcortical volumes, full‐scale IQ score, and MABC‐2 total score, with age, sex, and TBV included as covariates, in the patient and control groups separately. To test whether there was any association, in patients, between qualitative injury scores on neonatal MRI and regional volumes at 6 to 8 years, we performed Wilcoxon rank‐sum tests between the regional volumes of patients with no injury (score = 0) and those of patients with any injury (score >0) for each injury category (white matter, BGT, PLIC, and cortex). In all tests, correction for false discovery rate was applied and *p* < 0.05 was considered significant. Statistical analysis was performed using IBM SPSS version 24 (IBM Corp., Armonk, NY, USA).

## RESULTS

### Recruitment

Study recruitment is summarized in Figure S1. We recruited 50 patients and 43 controls for the study. Of these, seven patients and four controls did not want to undergo scanning and four patients had incomplete scans due to movement during the scan. Scans from eight patients and seven controls were not of sufficient quality because of movement artefacts. The remaining 31 patients and 32 controls were included in the analysis. The demographics in Table 1 show that controls were matched to patients for age, sex, and socioeconomic status and that there was no patient–control difference in head circumference. The patient group had reduced full‐scale IQ score and a larger proportion of individuals at risk of motor impairment compared with the control group. Detailed analyses of patient–control differences in domains of the cognitive and motor assessments have been previously reported for this cohort,^8^, ^9^ and for a cohort that overlapped with 15 patients and 14 controls included in this cohort.^7^, ^11^ The demographics, outcome scores, and perinatal clinical information of children excluded from the patient group because of incomplete or poor quality scans were not significantly different from those included in the analysis, apart from Apgar score (Table 1). Median Apgar score was higher in rejected patients than included patients (*p* = 0.040), indicating that the included cohort on average was in poorer condition at 10 minutes of age.

**TABLE 1:** Participants' demographics

	Patients (*n* = 31)	Controls (*n* = 32)	*p*	Rejected patients (*n* = 19)	*p*
**Childhood MRI**					
Age, years:months, median (range)	7:0 (6:0–7:11)	7:2 (6:1–7:10)	0.994	6:11 (6:2–7:11)	0.659
Sex, male/female	17/14	16/16	0.802	11/8	1.0
Index of multiple deprivation, median (range)	7 (1–10)	7 (3–10)	0.371	7 (2–9)	0.864
Head circumference (cm), median (range)	52.3 (47.2–57.5)	52.5 (48.5–56.5)	0.440	52.0 (50.3–56.5)	0.741
Full‐scale IQ, median (range)	97 (62–123)	109 (88–137)	<0.001	89 (81–107)	0.293
MABC‐2 total score, median (range)	11 (3–19)	11 (5–16)	0.643	8.5 (1–15)	0.182
MABC‐2 total score < 15th centile, *n* (%)	10 (32)	2 (6)	0.011	5 (26)	0.757
**Neonatal MRI scores**					
White matter					0.891
0	8			5	
1	13			5	
2	8			7	
3	2			1	
BGT					0.124
0	27			13	
1	3			5	
2	1			1	
3	0			0	
PLIC					0.106
0	28			14	
1	3			3	
2	0			2	
Cortex					0.563
0	19			13	
1	9			5	
2	1			1	
3	2			0	
**Perinatal clinical information**					
Assisted ventilation at 10 minutes of age, *n* (%)	24 (77)			11 (58)	0.205
Cardiac compressions required, *n* (%)	9 (29)			6 (32)	1.0
Apgar score at 10 minutes of age, median (range)	6 (0–10)			7 (2–10)	0.040
Worst pH within 1 hour of birth, median (range)	6.94 (6.70–7.25)			6.90 (6.65–7.34)	0.453
Amplitude‐integrated EEG abnormalities before therapeutic hypothermia: moderate/severe	29/2			18/1	1.0

Perinatal clinical information and Rutherford scores^17^ from neonatal MRI assessment of BGT (score 0–3), white matter (score 0–3), posterior limbs of the internal capsule (score 0–2), and cortex (score 0–3), are given for patients. Also shown are demographics of the rejected patients, and *p*‐values for the comparison between included and rejected patients. The *p*‐values were calculated with Fisher's exact tests or Wilcoxon rank‐sum tests. Abbreviations: BGT, basal ganglia and thalami; EEG, electroencephalogram; MABC‐2, Movement Assessment Battery for Children, Second Edition; MRI, magnetic resonance imaging; PLIC, posterior limb of internal capsule.

### Regional brain volumes

**FIGURE 2:**
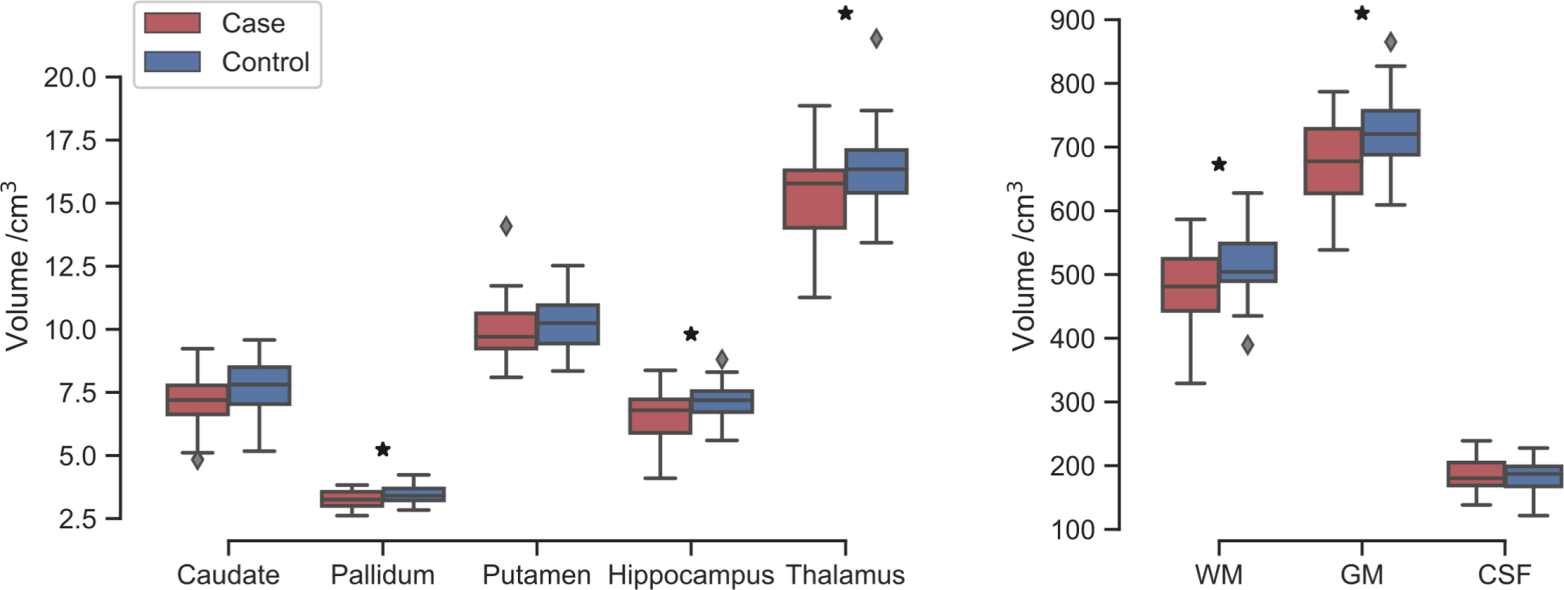
Patient–control comparison of regional volumes, for subcortical structures (left) and whole‐brain volumes, including white matter (WM), total cortical and subcortical grey matter (GM), and cerebrospinal fluid (CSF). Boxes indicate the interquartile range, with a line for the median, with whiskers extending to the range of the data and outliers shown as diamonds. Group statistics are shown in Table S2.

Figure 2 shows the distribution of regional brain volumes measured from MRI at 6 to 8 years, with statistics shown in Table S2. Patients had a reduced whole‐brain volume of grey matter (*p* = 0.003) and white matter (*p* = 0.026). Additionally, patients compared with controls had significantly lower volumes of pallidi (*p* = 0.026), hippocampi (*p* = 0.004), and thalami (*p* = 0.013). When taking differences in TBV into account, there were no significant differences in the volumes of subcortical structures (uncorrected *p* > 0.05; one‐way ANCOVA).

### Association with functional outcome

**FIGURE 3:**
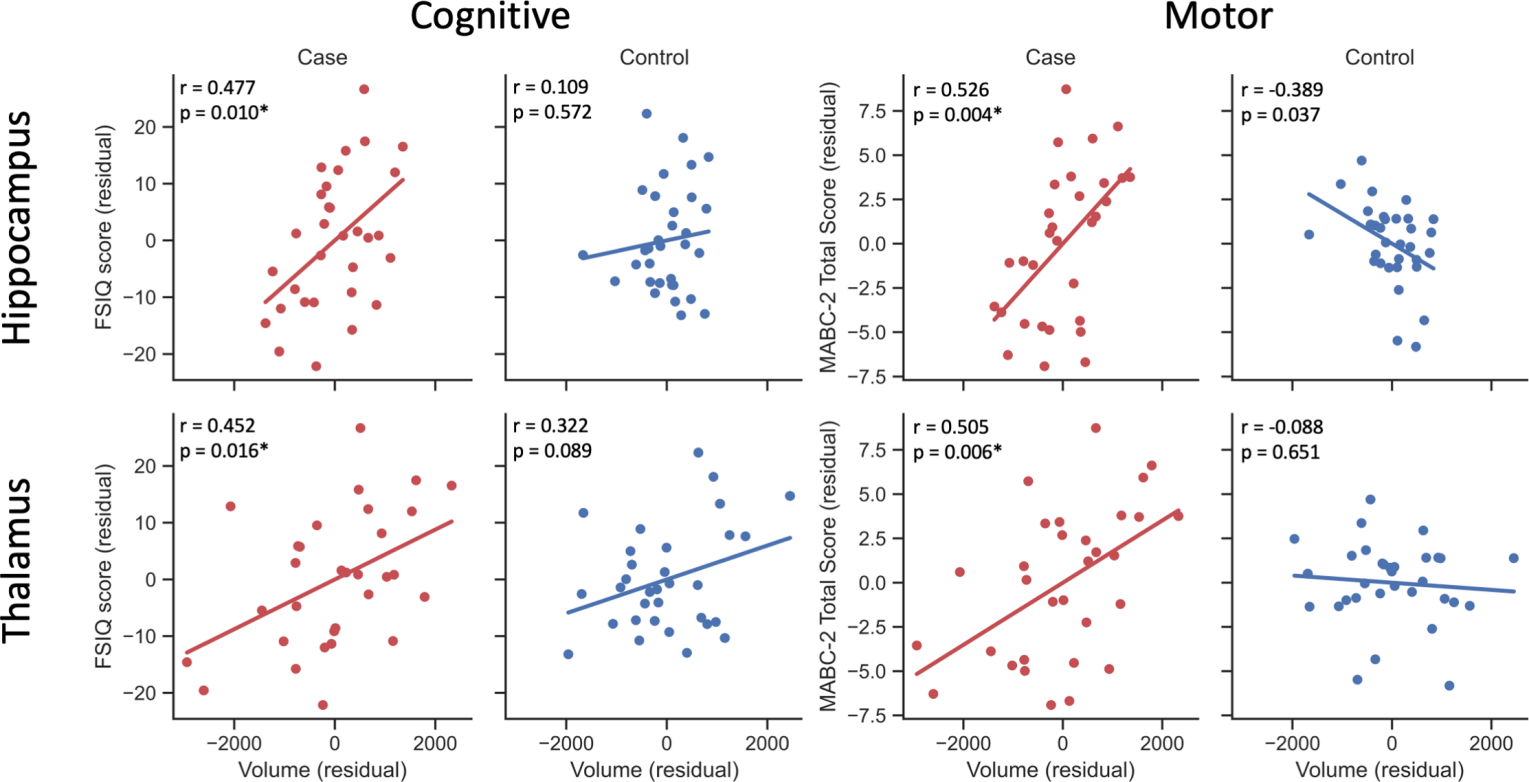
Correlation between subcortical volumes and cognitive and motor scores. Partial Pearson correlations were measured between volumes and outcome, in patients and controls separately, for both Movement Assessment Battery for Children, Second Edition (MABC‐2) total score and full‐scale IQ (FSIQ) score, with age, sex, and total brain volume included as covariates. Hippocampus and thalamus gave significant correlations with both FSIQ score and MABC‐2 total score in patients (red). Controls are shown for comparison (blue), although no correlations were significant after correction for false discovery rate. Each plot shows the residuals of subcortical volume against the residuals of functional outcome score, with a line showing the regression. Correlation coefficients and uncorrected *p*‐values are shown on each plot. *False discovery rate‐corrected *p* < 0.05.

Correlations between subcortical volumes and functional outcome are shown in Figure 3. In patients, full‐scale IQ score significantly correlated with the volume of the hippocampi (*r* = 0.477, *p* = 0.010) and thalami (*r* = 0.452, *p* = 0.016). Additionally, MABC‐2 total score significantly correlated with the volume of the hippocampi (*r* = 0.526, *p* = 0.004) and thalami (*r* = 0.505, *p* = 0.006). In controls, MABC‐2 total score correlated with the volume of the putamen (*r* = −0.378, *p* = 0.043) and hippocampi (*r* = −0.389, *p* = 0.037), but neither of these was significant after correction for false discovery rate .

### Association with neonatal injury scores

Median qualitative scores of injury on neonatal MRI in patients were low, as expected for this group, which did not include those with severe disability or any CP. There was no association between regional volumes at school‐age and qualitative injury scores on neonatal MRI for the white matter, BGT, or PLIC (false discovery rate‐corrected *q* > 0.05); however, the sample sizes for these injury sites were very small. Patients with cortical injury scores greater than 0 on neonatal MRI had significantly reduced hippocampi (*p* = 0.001), thalami (*p* = 0.002), grey matter (*p* = 0.015), and white matter (*p* = 0.013) compared with patients with no cortical injury. When TBV was included as a covariate in an ANCOVA, hippocampal volume was still associated with cortical injury (*p* = 0.002), but the association with thalamic volume was not significant after correction for false discovery rate. Regional brain volumes at school‐age are plotted against scores from qualitative scores of injury on neonatal MRI in Figures [Link], [Link], with statistics shown in Tables [Link], [Link].

## DISCUSSION

In this study, we demonstrated that early school‐age children who underwent therapeutic hypothermia for HIE, without CP, had reduced volumes of grey and white matter compared with matched controls. Additionally, patients had reduced volumes of pallidi, hippocampi, and thalami, which were not independent of TBV. We also demonstrated that, in cooled children, both full‐scale IQ score and MABC‐2 total score correlated with hippocampal volumes and thalamic volumes, independent of age, sex, and TBV. No significant correlations were found in controls. We found that patients with any cortical injury visible on neonatal MRI had reduced volumes of hippocampi, thalami, and grey and white matter at school‐age compared with patients with no cortical injury.

Previous studies have investigated the effect of HIE on brain volume. An early study using head circumference demonstrated that infants with HIE with only mild or moderate BGT lesions did not have abnormal head growth compared with controls,^26^ consistent with our cohort in which there were no differences in head circumference between patients and controls. An investigation of brain volume in young adults with a history of mild or moderate of HIE, before widespread use of therapeutic hypothermia, showed significant reduction in whole‐brain volume.^14^ Our study demonstrates that, in a population cooled for moderate to severe HIE, without CP, there is a reduction in whole‐brain grey‐ and white‐matter volume at school‐age compared with matched controls, despite no differences in head circumference.

Studies involving qualitative assessment of MRI scans on children with HIE, before the widespread use of therapeutic hypothermia, have demonstrated persistent abnormalities in the white matter and basal ganglia on MRI scans at 12 to 24 months^27^ and 9 to 10 years.^28^ Additionally, the hippocampus is known to be vulnerable to hypoxic injury.^13^, ^29^, ^30^ We found that the pallidi, hippocampi, and thalami were smaller at age 6 to 8 years in patients compared with controls. Lower hippocampal volume in patients may affect cognitive outcomes, and thus may be associated with the reduced perceptual reasoning, verbal comprehension, working memory, and full‐scale IQ scores in this population.^7^, ^8^ Similarly, lower thalamic volume in patients may affect fine or complex motor skills, as seen in this population, with patients having lower manual dexterity, aiming and catching, and MABC‐2 total score than matched controls.^7^ Although these absolute differences were not independent of TBV, the correlations of both full‐scale IQ and MABC‐2 total scores with hippocampal and thalamic volumes show that there was still variation in subcortical grey‐matter volumes within the patient group that was significantly associated with functional outcome, independent of TBV, age, and sex. The lack of significant reduction in subcortical grey‐matter volumes beyond those associated with an overall reduction in brain volume may have been due to our cohort comprising children without CP; the median qualitative score of injury on neonatal MRI was low, possibly resulting in small differences in regional brain volumes which were masked by normalization with TBV.

Hippocampal volume has been associated with IQ both in adults^31^ and in children.^32^ Neuroimaging studies have indicated that hippocampal damage sustained from perinatal asphyxia persists throughout development, resulting in memory difficulties and reduced IQ.^13^, ^33^ Additionally, thalamic volume has been shown to be associated with verbal IQ in typically developing children,^34^ and with visual memory performance in patients aged 14 to 25 years with developmental amnesia resulting from hippocampal atrophy caused by hypoxic‐ischaemic injury.^29^ In our control group, volumes of the hippocampi and thalami did not correlate with cognitive and motor scores. This may have been due to the small sample size, and could indicate that functional outcome in these children was dependent on a much larger number of factors. In the patient group of a similar size, volumes of the hippocampi and thalami correlated with both full‐scale IQ and MABC‐2 total score, suggesting stronger associations in this group.

Previous qualitative MRI studies of children with HIE have shown that injury patterns on neonatal MRI (most commonly in the BGT) are associated with later adverse neurodevelopmental outcomes.^15^ We found that volumes of hippocampi, thalami, grey matter, and white matter at school‐age were reduced in patients, with signal change indicating cortical injury on neonatal MRI compared with those with normal cortical signal. The association between neonatal cortical injury and hippocampal volume was independent of TBV, suggesting this association is due to those with abnormal cortical signal also having underlying primary injury to other structures which were not assessed during the neonatal qualitative assessment (e.g. the hippocampus); if the reduced school‐age hippocampal volume was secondary to cortical injury, we would expect it to be associated with the reduced grey‐ and white‐matter volumes (and therefore TBV) in these children. Despite therapeutic hypothermia there is still an effect of HIE on hippocampal volume which is probably related to neonatal hippocampal injury. Thus, therapeutic hypothermia may not be neuroprotective for hippocampal injury; animal studies have shown that development of the hippocampal GABAergic system is impaired despite therapeutic hypothermia.^35^

We found no association between regional brain volumes at school‐age and injury patterns in the BGT, PLIC, or white matter on neonatal MRI. This may suggest cortical injury is more sensitive than other sites of injury in contributing to altered structural brain development in cooled children without severe disability; however, owing to the small sample sizes in these tests, particularly for the PLIC, BGT, and white‐matter associations, we cannot draw firm conclusions from these analyses.

Owing to the difficulties associated with scanning children of this age group, the MRI data from many participants were not of sufficient quality to be included in the analysis. A larger sample could have enabled more sensitive detection of group differences and correlations, particularly as this cohort represented those with non‐severe outcomes and may therefore be characterized by very subtle differences in brain structure. Specifically, a larger cohort would allow more robust analysis of differences in regional brain volumes between patients with and without injury on neonatal MRI. Additionally, to increase statistical power and reduce the effect of multiple comparisons, we did not investigate associations with subscales of the cognitive and motor assessments. This is largely because few studies have investigated subcortical grey‐matter volumes in cooled children with non‐severe outcomes, so we opted for a more exploratory, rather than hypothesis‐driven, approach. Future studies could focus on the involvement of the hippocampus and thalamus in domains of cognitive and motor performance.

## CONCLUSION

We have demonstrated that early school‐age children cooled for HIE, without CP, had altered regional brain volumes compared with matched controls. Further investigation is required to determine neuroprotective strategies or therapeutic interventions that improve outcomes.

## Funding information

The Baily Thomas Charitable Fund (TRUST/VC/AC/SG4681‐7596), David Telling Charitable Trust, Sparks (05/BTL/01 and 14/BTL/01), and Moulton Foundation. A.P.C.S. is supported by the Wellcome Trust (WT220070/Z/20/Z). J.C.W.B. is supported by the UK Medical Research Council (MR/N026969/1).

## Supporting information


**Table S1:** Criteria for qualitative assessment of the presence and extent of brain injury on neonatal MRI.

**Table S2:** Regional volume for patients and controls.

**Table S3:** Regional volume at school‐age for patients grouped by white matter injury scores on neonatal MRI.

**Table S4:** Regional volume at school‐age for patients grouped by basal ganglia and thalamus injury scores on neonatal MRI.

**Table S5:** Regional volume at school‐age for patients grouped by posterior limb of internal capsule injury scores on neonatal MRI.

**Table S6:** Regional volume at school‐age for patients grouped by cortex injury scores on neonatal MRI.

**Figure S1:** Study recruitment.

**Figure S2:** Plots showing the distribution of volumes, measured from MRI at 6–8 years, with patients grouped by scores from neonatal MRI assessment of white matter.

**Figure S3:** Plots showing the distribution of volumes, measured from MRI at 6–8 years, with patients grouped by scores from neonatal MRI assessment of basal ganglia and thalami.

**Figure S4:** Plots showing the distribution of volumes, measured from MRI at 6–8 years, with patients grouped by scores from neonatal MRI assessment of posterior limbs of the internal capsule.

**Figure S5:** Plots showing the distribution of volumes, measured from MRI at 6–8 years, with patients grouped by scores from neonatal MRI assessment of the cortex.

## Data Availability

The data that support the findings of this study are available from the corresponding author upon reasonable request.
