# Supplementary material for: Brain volumes and functional outcomes in children without cerebral palsy after therapeutic hypothermia for neonatal hypoxic‐ischaemic encephalopathy
Source: Dev Med Child Neurol. 2022 Jul 30;65(3):367–75. doi: 10.1111/dmcn.15369 (PMC10087533; doi:10.1111/dmcn.15369)
Supplement: Supplementary file 1 — Table S1: Criteria for qualitative assessment of the presence and extent of brain injury on neonatal MRI. [file DMCN-65-367-s010.docx]

| Injury Site | Score | Description |
| --- | --- | --- |
| Posterior limb of internal capsule (PLIC) | 0 | Normal |
|  | 1 | Equivocal – reduced or asymmetrical signal intensity |
|  | 2 | Loss – reversed or abnormal signal intensity bilaterally on T1 and/or T2 |
| Basal ganglia and thalami (BGT) | 0 | Normal |
|  | 1 | Mild – focal abnormal signal intensity |
|  | 2 | Moderate – multifocal abnormal signal intensity |
|  | 3 | Severe – widespread abnormal signal intensity |
| White matter (WM) | 0 | Normal |
|  | 1 | Mild – exaggerated long T1 and long T2 in periventricular white matter only |
|  | 2 | Moderate – long T1 and long T2 extending out to subcortical white matter and/or focal punctate lesions or focal area of infarction |
|  | 3 | Severe – widespread abnormalities including overt infarction, haemorrhage, and long T1 and long T2 |
| Cortex |  | Scored as the presence of abnormal signal intensity, usually decreased T1 or cortical highlighting. Specifically, sites included the central sulcus, interhemispheric fissure, insula, and occipital cortex. |
|  | 0 | Normal |
|  | 1 | Mild – 1–2 sites involved |
|  | 2 | Moderate – 3 sites involved |
|  | 3 | Severe – more than 3 sites involved. |

Supplementary Table 1: Criteria for qualitative assessment of the presence and extent of brain injury on neonatal MRI, as defined by Rutherford et al., 2010.
