# Supplementary material for: Brain volumes and functional outcomes in children without cerebral palsy after therapeutic hypothermia for neonatal hypoxic‐ischaemic encephalopathy
Source: Dev Med Child Neurol. 2022 Jul 30;65(3):367–75. doi: 10.1111/dmcn.15369 (PMC10087533; doi:10.1111/dmcn.15369)
Supplement: Supplementary file 2 — Table S2: Regional volume for patients and controls. [file DMCN-65-367-s001.docx]

|  | Cases (n = 31) | Controls (n = 32) | 95% CI | p |
| --- | --- | --- | --- | --- |
| Caudate | 7166 (1045) | 7663 (1113) | -1084.1, 1.3 | 0.073 |
| Pallidum | 3248 (347) | 3452 (361) | -389.2, -36.1 | 0.026* |
| Putamen | 9959 (1157) | 10328 (1195) | -1019.1, 169.4 | 0.219 |
| Hippocampus | 6486 (1165) | 7192 (672) | -1181.1, -242.1 | 0.004* |
| Thalamus | 15222 (2017) | 16362 (1490) | -2061.0, -300.2 | 0.013* |
| Grey matter | 677092 (65041) | 725883 (57890) | -81399.5, -19954.0 | 0.003* |
| White matter | 482011 (60864) | 513920 (49871) | -61515.5, -5960.6 | 0.026* |
| CSF | 185569 (25905) | 184224 (23807) | -11068.9, 13580.6 | 0.831 |

Supplementary Table 2: Regional volume for cases and controls, displayed as mean (standard deviation) in mm^3^. Also shown are the 95% confidence intervals (CI) of the difference between means, and the p-value from a two-tailed t-test. *FDR-corrected q<0.05.
