# Supplementary material for: Brain volumes and functional outcomes in children without cerebral palsy after therapeutic hypothermia for neonatal hypoxic‐ischaemic encephalopathy
Source: Dev Med Child Neurol. 2022 Jul 30;65(3):367–75. doi: 10.1111/dmcn.15369 (PMC10087533; doi:10.1111/dmcn.15369)
Supplement: Supplementary file 3 — Table S3: Regional volume at school‐age for patients grouped by white matter injury scores on neonatal MRI. [file DMCN-65-367-s006.docx]

|  | Cases with WM injury score = 0 (n = 8) | Cases with WM injury scores >0 (n = 23) | p |
| --- | --- | --- | --- |
| Caudate | 7123 (956) | 7219 (1559) | 1.0 |
| Pallidum | 3304 (529) | 3224 (630) | 0.527 |
| Putamen | 9935 (643) | 9652 (1571) | 0.718 |
| Hippocampus | 6990 (1657) | 6384 (2129) | 0.240 |
| Thalamus | 15696 (2402) | 15797 (3441) | 0.367 |
| Grey matter | 669756 (81282) | 685639 (125039) | 0.928 |
| White matter | 475172 (87326) | 481212 (90389) | 0.786 |
| CSF | 188786 (43837) | 176395 (35764) | 0.892 |

Supplementary Table 3: Regional volume at school age for cases grouped by white matter (WM) injury scores on neonatal MRI, displayed as median (IQR) in mm^3^. Also shown are p-values from Wilcoxon rank sum tests.
