# Supplementary material for: Brain volumes and functional outcomes in children without cerebral palsy after therapeutic hypothermia for neonatal hypoxic‐ischaemic encephalopathy
Source: Dev Med Child Neurol. 2022 Jul 30;65(3):367–75. doi: 10.1111/dmcn.15369 (PMC10087533; doi:10.1111/dmcn.15369)
Supplement: Supplementary file 4 — Table S4: Regional volume at school‐age for patients grouped by basal ganglia and thalamus injury scores on neonatal MRI. [file DMCN-65-367-s007.docx]

|  | Cases with BGT injury score = 0 (n = 27) | Cases with BGT injury scores >0 (n = 4) | p |
| --- | --- | --- | --- |
| Caudate | 7219 (1204) | 6634 (3282) | 0.480 |
| Pallidum | 3254 (528) | 2815 (938) | 0.157 |
| Putamen | 9846 (1447) | 9194 (2276) | 0.480 |
| Hippocampus | 6937 (1410) | 5619 (2697) | 0.175 |
| Thalamus | 15797 (2246) | 12733 (6382) | 0.239 |
| Grey matter | 677758 (97886) | 677444 (207915) | 0.860 |
| White matter | 481212 (83294) | 478722 (215201) | 0.906 |
| CSF | 180252 (40405) | 192143 (67070) | 1.0 |

Supplementary Table 4: Regional volume at school age for cases grouped by basal ganglia and thalamus (BGT) injury scores on neonatal MRI, displayed as median (IQR) in mm^3^. Also shown are p-values from Wilcoxon rank sum tests.
