# Supplementary material for: Brain volumes and functional outcomes in children without cerebral palsy after therapeutic hypothermia for neonatal hypoxic‐ischaemic encephalopathy
Source: Dev Med Child Neurol. 2022 Jul 30;65(3):367–75. doi: 10.1111/dmcn.15369 (PMC10087533; doi:10.1111/dmcn.15369)
Supplement: Supplementary file 5 — Table S5: Regional volume at school‐age for patients grouped by posterior limb of internal capsule injury scores on neonatal MRI. [file DMCN-65-367-s003.docx]

|  | Cases with PLIC injury score = 0 (n = 28) | Cases with PLIC injury scores >0 (n = 3) | p |
| --- | --- | --- | --- |
| Caudate | 7209 (1179) | 6636 (-) | 0.738 |
| Pallidum | 3253 (551) | 2932 (-) | 0.548 |
| Putamen | 9775 (1445) | 9488 (-) | 0.894 |
| Hippocampus | 6865 (1412) | 6172 (-) | 0.423 |
| Thalamus | 15789 (2302) | 14100 (-) | 0.738 |
| Grey matter | 674226 (99340) | 748925 (-) | 0.593 |
| White matter | 480779 (80840) | 523102 (-) | 0.640 |
| CSF | 182909 (38342) | 175240 (-) | 0.462 |

Supplementary Table 5: Regional volume at school age for cases grouped by posterior limb of internal capsule (PLIC) injury scores on neonatal MRI, displayed as median (IQR) in mm^3^. Note the IQR is not given for the cases with injury scores >0 due to the sample size. Also shown are p-values from Wilcoxon rank sum tests.
