# Supplementary material for: Brain volumes and functional outcomes in children without cerebral palsy after therapeutic hypothermia for neonatal hypoxic‐ischaemic encephalopathy
Source: Dev Med Child Neurol. 2022 Jul 30;65(3):367–75. doi: 10.1111/dmcn.15369 (PMC10087533; doi:10.1111/dmcn.15369)
Supplement: Supplementary file 6 — Table S6: Regional volume at school‐age for patients grouped by cortex injury scores on neonatal MRI. [file DMCN-65-367-s009.docx]

|  | Cases with cortex injury score = 0 (n = 19) | Cases with cortex injury scores >0 (n = 12) | p |
| --- | --- | --- | --- |
| Caudate | 7263 (1091) | 6703 (2236) | 0.081 |
| Pallidum | 3336 (470) | 3016 (552) | 0.043 |
| Putamen | 10218 (1349) | 9376 (1121) | 0.035 |
| Hippocampus | 7044 (1141) | 5259 (1752) | 0.001* |
| Thalamus | 16286 (1618) | 13476 (3934) | 0.002* |
| Grey matter | 693113 (83515) | 638963 (83884) | 0.015* |
| White matter | 503941 (94978) | 446392 (67267) | 0.013* |
| CSF | 194422 (47345) | 176332 (20133) | 0.598 |

Supplementary Table 6: Regional volume at school age for cases grouped by cortex injury scores on neonatal MRI, displayed as median (IQR) in mm^3^. Also shown are p-values from Wilcoxon rank sum tests. *FDR-corrected q<0.05.
