# Supplementary figures and images for: Brain volumes and functional outcomes in children without cerebral palsy after therapeutic hypothermia for neonatal hypoxic‐ischaemic encephalopathy
Source: Dev Med Child Neurol. 2022 Jul 30;65(3):367–75. doi: 10.1111/dmcn.15369 (PMC10087533; doi:10.1111/dmcn.15369)

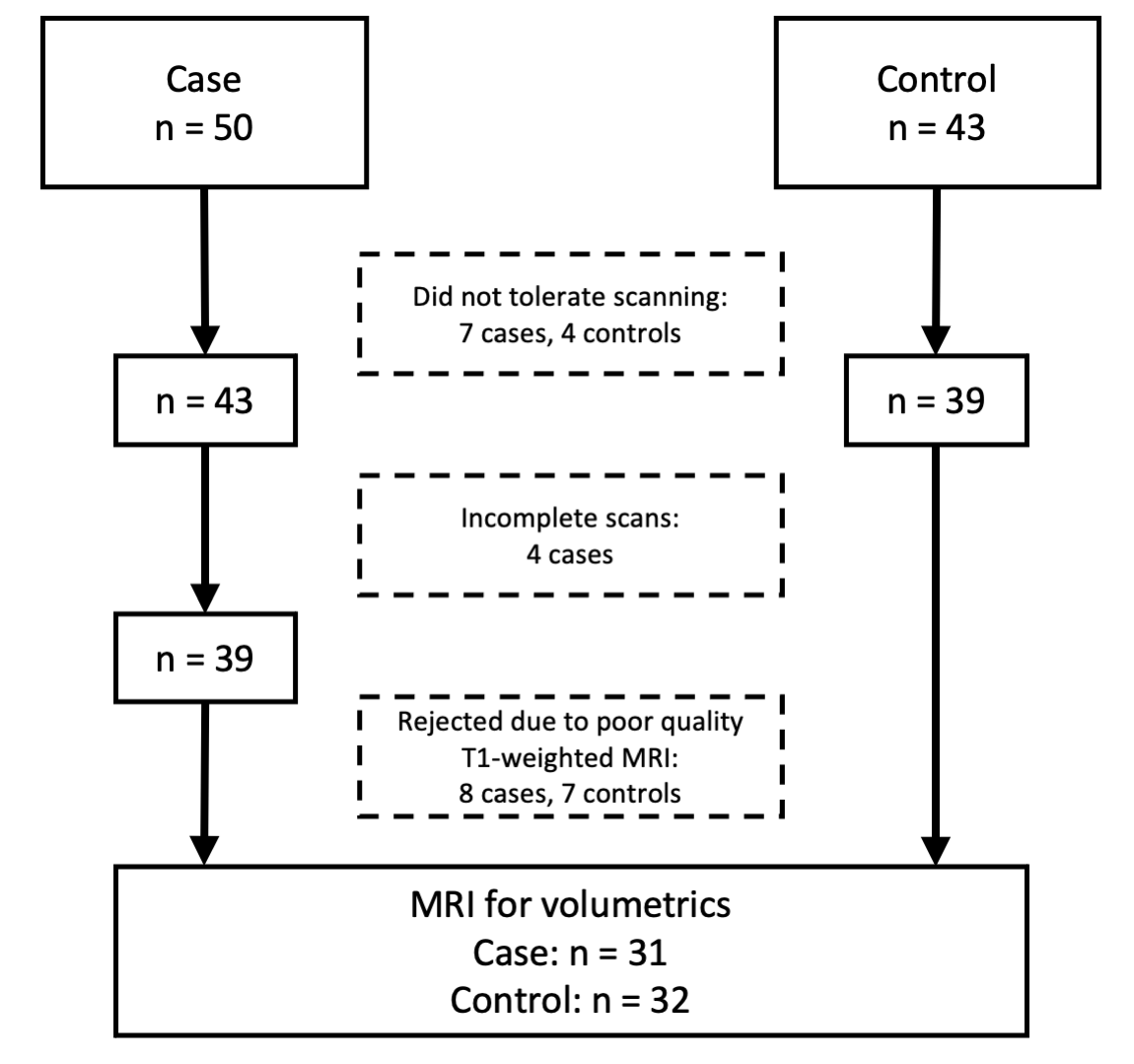

Supplement: Supplementary file 7 — Figure S1: Study recruitment. [file DMCN-65-367-s002.tiff]

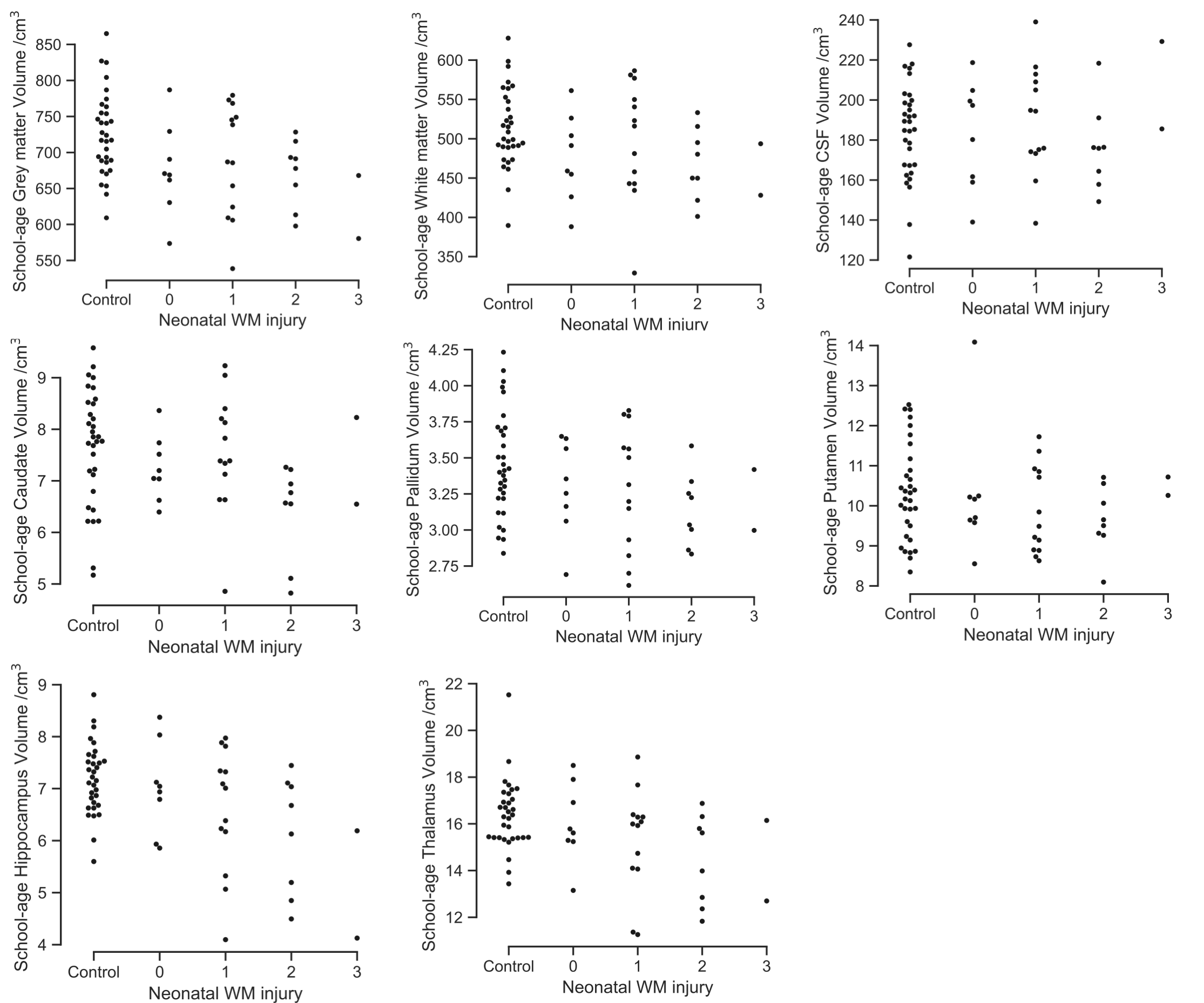

Supplement: Supplementary file 8 — Figure S2: Plots showing the distribution of volumes, measured from MRI at 6–8 years, with patients grouped by scores from neonatal MRI assessment of white matter. [file DMCN-65-367-s011.tiff]

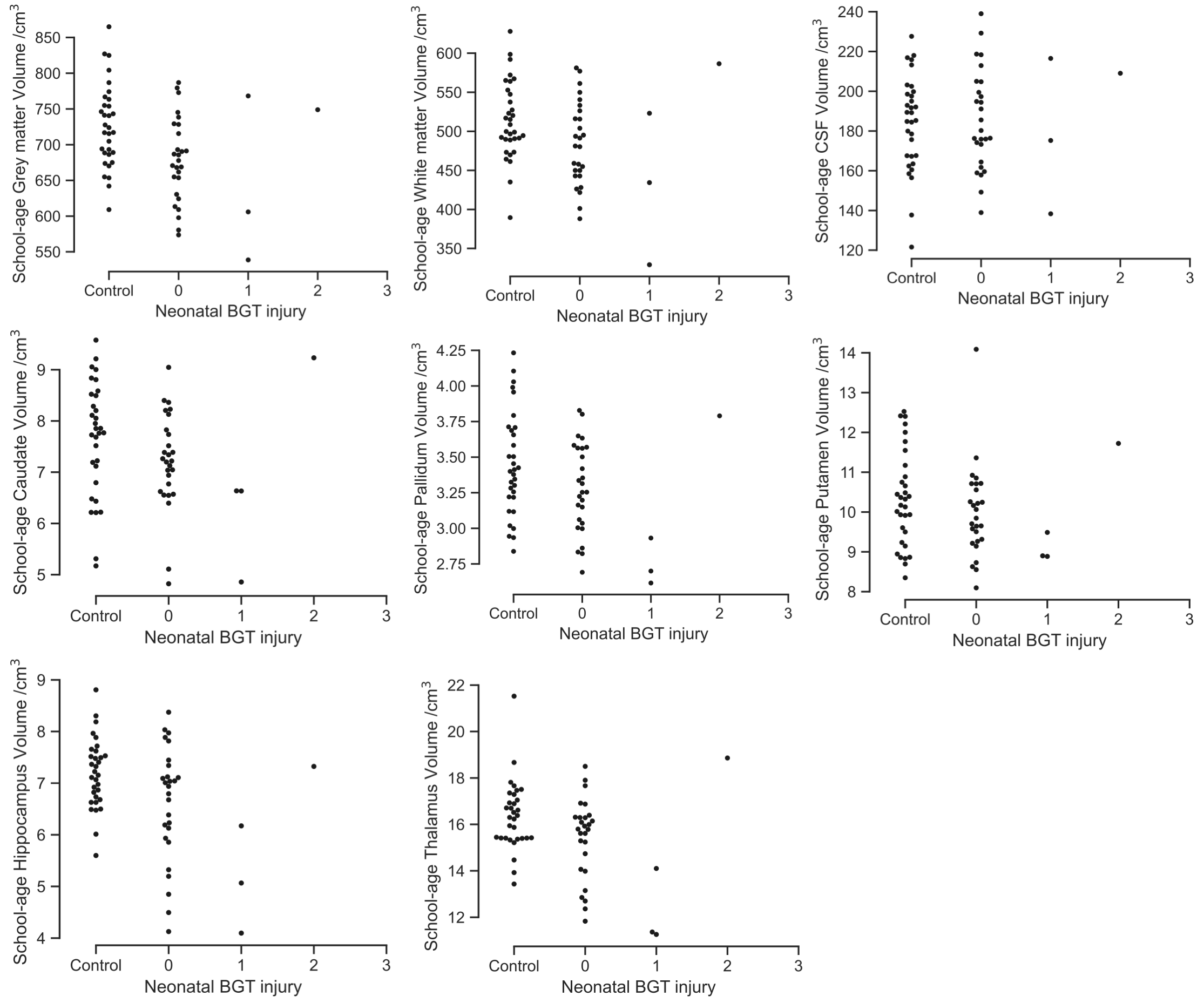

Supplement: Supplementary file 9 — Figure S3: Plots showing the distribution of volumes, measured from MRI at 6–8 years, with patients grouped by scores from neonatal MRI assessment of basal ganglia and thalami. [file DMCN-65-367-s005.tiff]

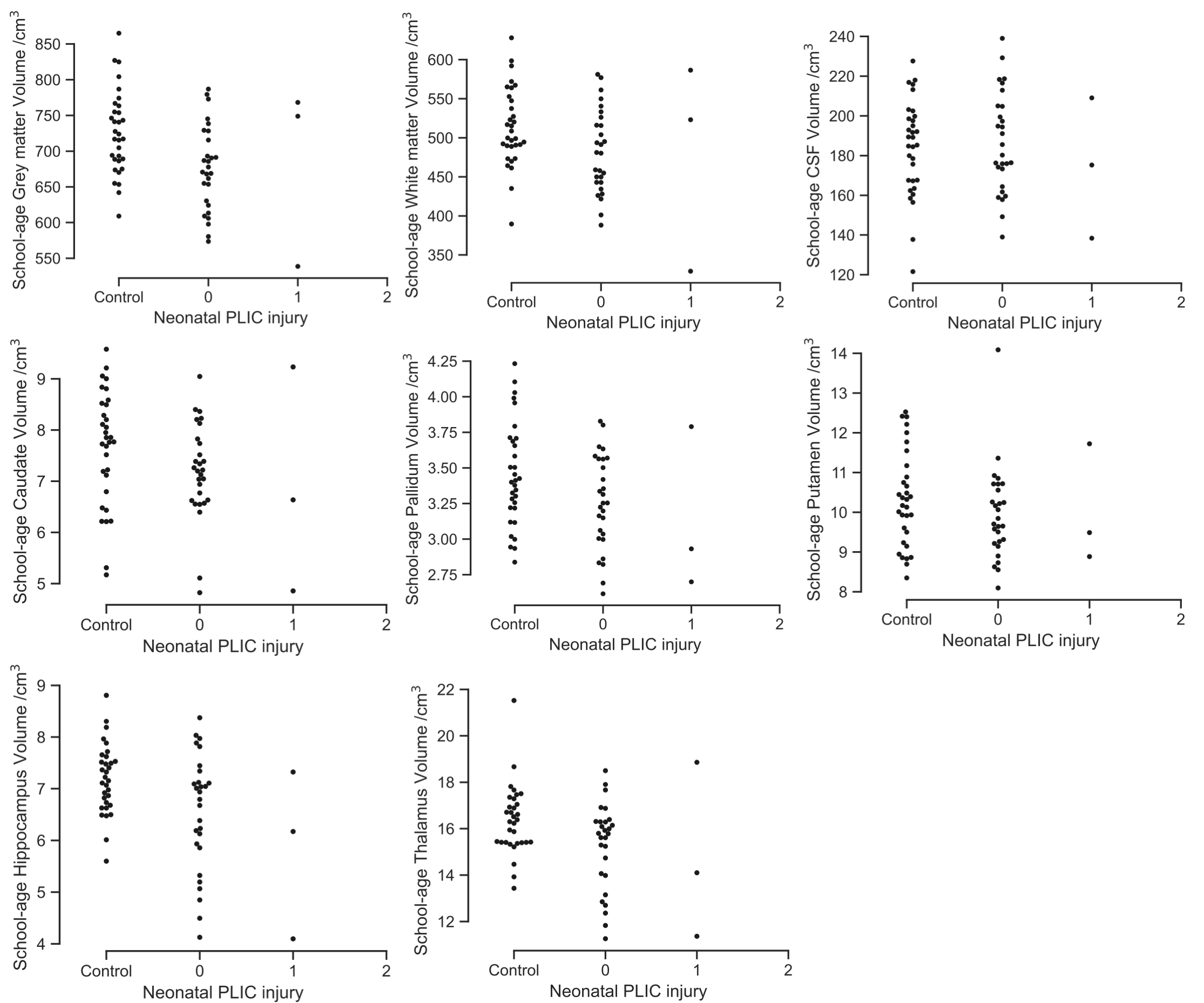

Supplement: Supplementary file 10 — Figure S4: Plots showing the distribution of volumes, measured from MRI at 6–8 years, with patients grouped by scores from neonatal MRI assessment of posterior limbs of the internal capsule. [file DMCN-65-367-s004.tiff]

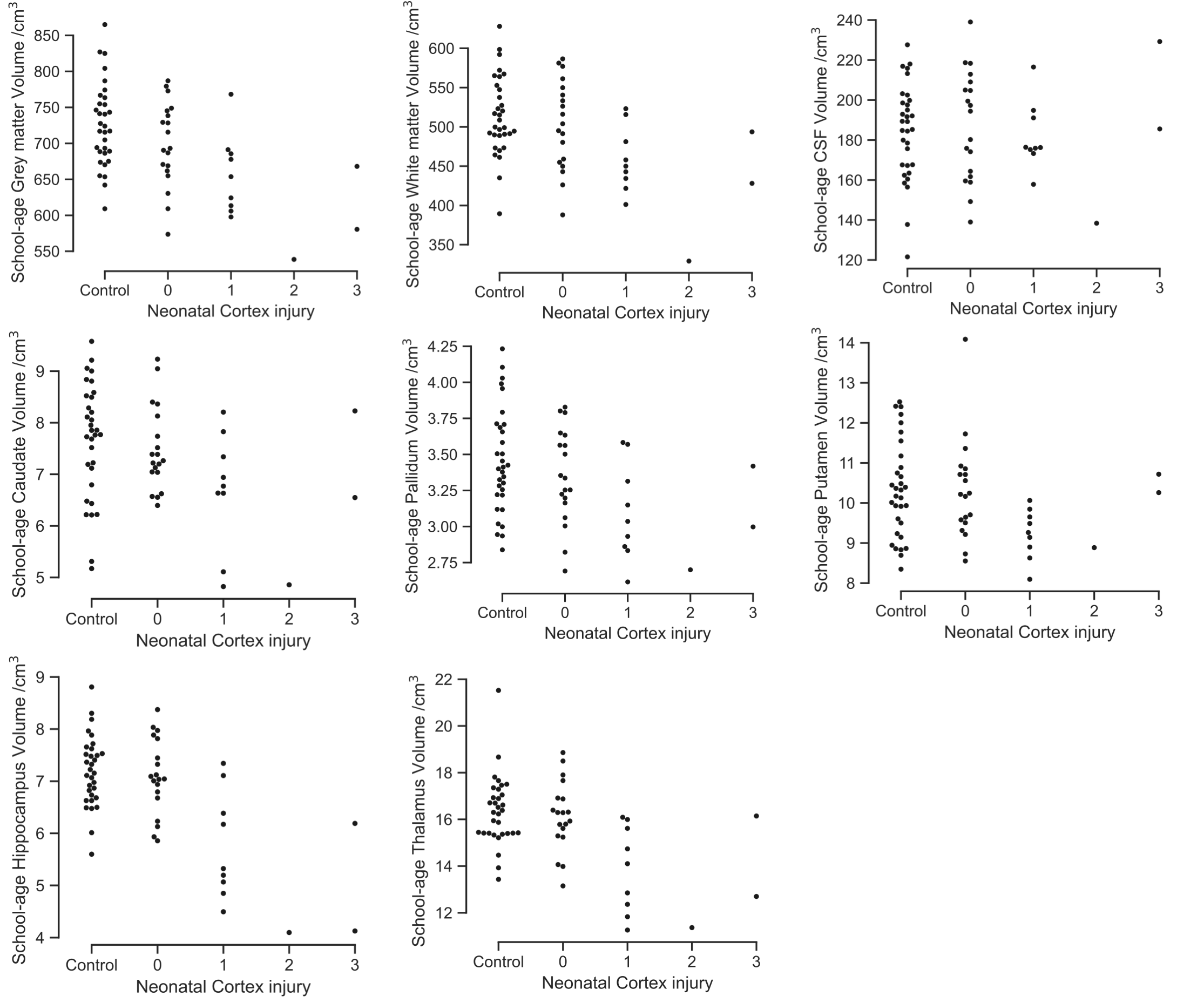

Supplement: Supplementary file 11 — Figure S5: Plots showing the distribution of volumes, measured from MRI at 6–8 years, with patients grouped by scores from neonatal MRI assessment of the cortex. [file DMCN-65-367-s008.tiff]
